# Prevalence and aggravation of cervical spine instabilities in rheumatoid arthritis during over 10 years: a prospective multicenter cohort study

**DOI:** 10.1038/s41598-024-78429-9

**Published:** 2024-11-05

**Authors:** Yutaro Kanda, Takashi Yurube, Hiroaki Hirata, Masatoshi Sumi

**Affiliations:** 1 Department of Orthopaedic Surgery, Kobe University Graduate School of Medicine, 7-5-1 Kusunoki-cho, Chuo-ku, Kobe, 650-0017 Japan; 2 Department of Orthopaedic Surgery, Hyogo Prefectural Harima-Himeji General Medical Center, 3-264 Kamiya-cho, Himeji, 670-8560 Japan; 3 Department of Orthopaedic Surgery, Kobe Rosai Hospital, 4-1-23 Kagoike-dori, Chuo-ku, Kobe, 651-0053 Japan; 4 Department of Orthopaedic Surgery, Mahoshi Hospital, 12-3 Yamada-cho Kamitanigami Aza Kokodani, Kita-ku, Kobe, 651-1242 Japan

**Keywords:** Rheumatoid arthritis (RA), Cervical spine instability, Atlantoaxial subluxation (AAS), Vertical subluxation (VS), Subaxial subluxation (SAS), Natural history and course, Musculoskeletal system, Rheumatology, Rheumatic diseases, Musculoskeletal abnormalities, Rheumatoid arthritis

## Abstract

We designed a prospective multicenter cohort study to clarify a long-term, > 10-year prevalence and aggravation of cervical spine instabilities in rheumatoid arthritis (RA). In 2001–2002, 634 outpatients were enrolled, and 233 (36.8%) were followed for > 10 years. Cervical spine instability was defined as atlantoaxial subluxation (AAS, > 3-mm atlantodental interval), vertical subluxation (VS, < 13-mm Ranawat value), and subaxial subluxation (SAS, ≥ 2-mm irreducible vertebral translation). The aggravation was determined as ≥ 2 mm decrease of the Ranawat value in VS, ≥ 2-mm increase of slippage in SAS, and these new developments. The prevalence of VS and SAS increased during both the initial and last > 5 years (all, *p* ≤ 0.049). While VS aggravation was associated with pre-existing AAS (*p* = 0.007) and VS (*p* = 0.002), SAS aggravation correlated with pre-existing VS (*p* = 0.002). Multivariable analysis found hand mutilating changes (odds ratio [OR] = 4.048, *p* = 0.008), RA duration ≥ 5 years (OR = 3.711, *p* = 0.013), C-reactive protein (CRP) level ≥ 3.8 mg/dL (OR = 2.187, *p* = 0.044), and previous joint surgery (OR = 2.147, *p* = 0.021) as predictors for VS aggravation. Pre-existing VS (OR = 2.252, *p* = 0.024) and CRP ≥ 1.0 mg/dL (OR = 2.139, *p* = 0.013) were disclosed as predictors for SAS aggravation. Low disease activity and clinical remission before the development of VS and advanced peripheral joint destruction are essential to prevent progressive cervical spine instability in RA.

## Introduction

Rheumatoid arthritis (RA) is a chronic autoimmune disorder with unknown etiology, affecting 0.5–1% of the adult population^1,2^. This disease arises chronic inflammation in the synovial joints in extremities and spine, which results in characteristic deformities and carries a substantial burden not only for the individual but also for the society^3^. The cervical spine is a particularly common involvement in RA, which can lead to three distinctive instabilities: atlantoaxial subluxation (AAS)^4–10^, vertical subluxation (VS) of the axis^11,12^, and subaxial subluxation (SAS)^13^. Each instability can progress over time alone or in combination of other instabilities. Cautiously, these lesions can cause intractable neck pain and irreversible neurological damage with non-ambulation, respiratory dysfunction, and even sudden death in the worst scenario due to the potential development of spinal cord, cranial nerve, brainstem, and vertebral artery compression^14^. It is thus fundamental to understand the natural history of cervical spine involvement including the new onset and aggravation of instabilities. However, many prior investigations have been limited to the cross-sectional or retrospective design during < 10 years^15^. Because of drastic changes in RA treatment, e.g. biological agents, more recent prevalence, progression pattern, and predictive risk factors of cervical spine instabilities based on a longer-period observation are necessary.

We started a prospective multicenter cohort study of cervical spine, hand, and finger involvement in established RA fulfilling the “classical” or “definite” criteria in 2001–2002^16,17^. At > 5-year radiographic examination in 2006–2008, 43.6% of patients without any cervical spine instabilities newly developed AAS, VS, and/or SAS with the increased prevalence of VS and SAS, which was further accelerated in those with pre-existing instability^18–20^. Thereafter, we further followed our RA cohort for a longer period of additional > 5 years. At > 10-year radiographic examination in 2012–2013, more plausible predictive risk factors for the development of severe cervical spine instabilities which could cause compression myelopathy were identified as mutilating changes in the hands, concomitant corticosteroid treatment, and previous peripheral joint surgery^21^. Meanwhile, treatment aiming at low disease activity and clinical remission was a key factor associated with the absence of instability^22^. However, the detailed natural course of instabilities for > 10 years were not the subject of previous studies. Therefore, the objective of the present study was to clarify the > 10-year prevalence and progression pattern—focusing on the aggravation—of cervical spine instabilities in patients with RA. 

## Methods

### Study design and ethics statement

The current prospective observational study was performed at 21 research facilities in Hyogo, Japan. The sample size calculation was not applicable because of the explorative design. This study was designed from the same data collection cohort as our previous report^21^.

The study protocol was approved by the institutional review board at each facility. Written informed consent was obtained from each patient at the study enrollment. The present study was conducted in accordance with the principles of the Declaration of Helsinki and the laws and regulations of Japan.

### Patients

**Fig. 1:**
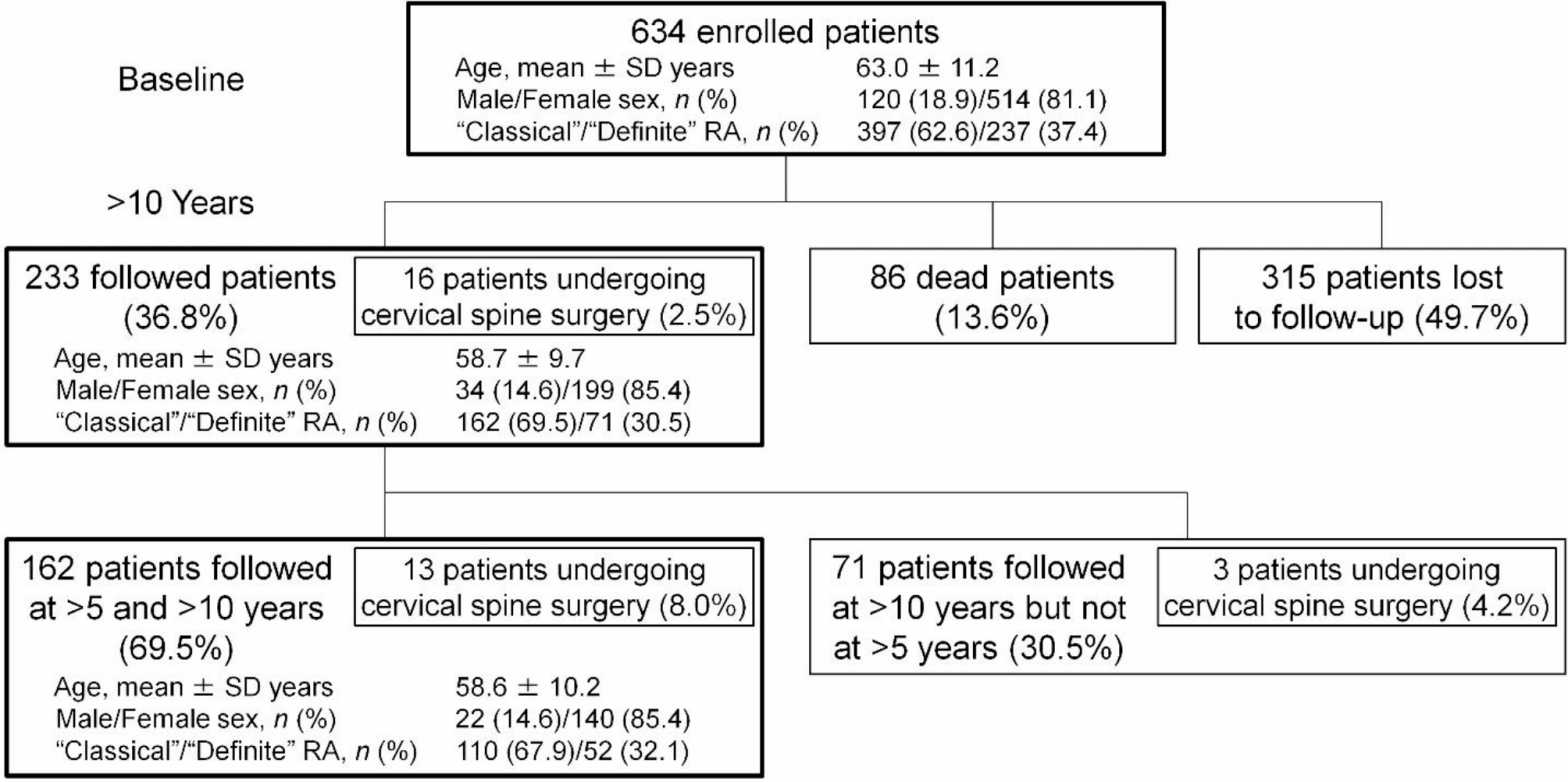
Number of outpatients, fulfilling the American Rheumatism Association 1958 criteria for “classical” or “definite” RA, enrolled at baseline and prospectively followed as outpatients, patients undergoing cervical spine surgery, dead patients, or patients lost to follow-up at > 5 and > 10 years. *RA* rheumatoid arthritis, *SD* standard deviation.

In 2001–2002, 634 Japanese outpatients who fulfilled the American Rheumatism Association 1958 criteria for ‘‘classical’’ or ‘‘definite’’ RA^16^ as well as the American College of Rheumatology 1987 revised criteria for RA^17^ were enrolled. Cervical spine instability was radiographically identified with the definition of three types. In 2012–2013, 233 (36.8%) were prospectively followed as outpatients every 3 months and radiographically assessed again after > 10 years. During the study period, 16 (6.9%) of 233 patients underwent cervical spine surgery, because of compression myelopathy in all cases. The observed myelopathy was associated with AAS alone (*n* = 5), VS alone (*n* = 1), SAS alone (*n* = 1), combined AAS and VS (*n* = 1), combined AAS and SAS (*n* = 1), combined VS and SAS (*n* = 3), or combined AAS, VS, and SAS (*n* = 4). The mean follow-up period was 11.0 ± 1.9 years. Letter and telephone survey revealed that 86 (13.6%) of 634 patients had died; thus, the final follow-up rate was 50.3% (Fig. 1).

Then, 162 (69.5%) of 233 patients had radiographic checkups both at > 5 (2006–2008) and > 10 (2012–2013) years, who were additionally analyzed to clarify time-course changes in the involvement of cervical spine instabilities. Of 162 patients, 13 (8.0%) received cervical spine surgery, due to myelopathy in all cases. The detected myelopathy was associated with AAS alone (*n* = 5), VS alone (*n* = 1), combined VS and SAS (*n* = 3), or combined AAS, VS, and SAS (*n* = 4). The mean follow-up period was 11.0 ± 0.3 years (Fig. 1).

### Radiographs

Lateral cervical spine radiographs were obtained in full-flexion, neutral, and full-extension positions under a standardized protocol (exposure time 80 ms, distance 150 cm, current 250 mA, and voltage 72 kV) with the placement of a scale marker^18–21^. Cervical spine instability was defined as three types: AAS with the anterior atlantodental interval (ADI) of > 3 mm^4,5^, VS with the Ranawat value of < 13 mm^11^, and SAS with irreducible vertebral translation of ≥ 2 mm without osteophyte formation^13^. To simplify the classification of instabilities, VS combined with AAS was categorized as VS. The aggravation was determined as follows: ≥2-mm decrease of the Ranawat value in pre-existing VS, ≥ 2 mm increase of vertebral slippage and/or at multiple segments in pre-existing SAS, and new development of VS and SAS. In addition, baseline C2–C7 angle was measured to evaluate the sagittal alignment of the cervical spine. Radiographs were measured twice at a 1-week interval by each of four rheumatologists blinded to this study. The prevalence and aggravation of cervical spine instabilities were set based on the average measurement values. The presence of pre-existing instabilities was included as explanatory variables for the aggravation of VS and SAS, based on a prior report^23^.

Bilateral hand radiographs providing views of the wrist, carpal, and fingers were taken to classify the severity of peripheral joint destruction into five categories: Steinbrocker classification stages I–IV^24^ and mutilating changes^25,26^. As previously established^26^, ≥ 3 of 10 ‘‘mutilans fingers’’ in the bilateral hands were considered mutilating changes^25^. Radiographs were reviewed twice at a 1-week interval by another three rheumatologists blinded to this study. Steinbrocker stages and mutilating changes were determined by majority decision. Baseline RA severity was extracted as explanatory variables for VS and SAS aggravation, since it clinically depends on the number of joints with erosion within the first 5–10 years^27^.

### Demographic, clinical, and disease characteristics

Demographic, clinical, and disease characteristics of patients were recorded at baseline. Age < 55 years^10,13^ and ≥ 65 years^28^, male sex^29^, RA duration ≥ 5 years^27^, and the presence of surgically treated peripheral joints for RA^28,30,31^ were marked as potential predictive risk factors of cervical spine instability. Serum C-reactive protein (CRP) level when ≥ 3.8 mg/dL, correlated with myelopathic deterioration^32^, or when > 1.0 mg/dL, indicating unsuccessful RA remission^33^, and the positivity for rheumatoid factor (RF), associated with subluxation^7,10^, were also listed. Thus, these were considered as explanatory variables for VS and SAS aggravation. Moreover, serum CRP level was measured again at > 10-year follow-up to evaluate time-course changes in disease activity during the study period.

### Treatments

To treat RA, patients received intensive use of disease-modifying antirheumatic drugs (DMARDs): methotrexate (MTX) (≤ 8 [1999– in Japan] and ≤ 16 [2011–] mg/week), salazosulfapyridine (≤ 1000 mg/day), D-penicillamine (≤ 100 mg/day), intramuscular gold (≤ 25 mg/2 weeks), and tacrolimus (1.5–3.0 mg/day [2005–]). Oral corticosteroids (≤ 10-mg/day prednisolone) were allowed to relieve RA symptoms. In more aggressive cases, biological agents such as infliximab (3 mg/kg/8 weeks [2003–]), etanercept (10–25 mg/twice a week [2005–]), adalimumab (40 mg/2 weeks [2008–]), tocilizumab (8 mg/kg/4 weeks [2008–]), abatacept (125 mg/week [2010–]), golimumab (50–100 mg/4 weeks [2011–]), and certolizumab (200 mg/2 weeks [2013–]) or a Janus kinase (JAK) inhibitor tofacitinib (5–10 mg/day [2013–]) were applied. Patients were categorized based on drug administration at baseline and throughout ≥ 5 years during > 10 years. Patients who had therapy with tacrolimus, biological agents, or tofacitinib for ≥ 3 years were similarly identified, because these drugs had not been approved for RA at baseline. Consequently, as no patients underwent ≥ 5-year treatment with tacrolimus or tofacitinib, the use of corticosteroids, MTX, other DMARDs, and biological agents was recorded as potential predictors for VS and SAS aggravation.

### Statistical analysis

Descriptive statistics for continuous variables are presented as the mean ± standard deviation, categorical variables as the frequency (percentages). The intraclass correlation coefficient (ICC) and Cohen’s kappa coefficient (κ) were calculated to assess the intraobserver and interobserver reliability for parametric and nonparametric radiographic parameters, respectively. In the entire cohort (*n* = 233), the prevalence of cervical spine instabilities at the baseline and endpoint of > 10-year follow-up was compared using the chi-squared test. In the subcohort (*n* = 162), the prevalence of instabilities at baseline, > 5-year follow-up, and > 10-year follow-up was also compared using the chi-squared test.

The chi-squared test for categorical variables and the Student’s *t*-test for continuous variables were used to assess differences between patients with and without the aggravation of VS and SAS at > 10-year follow-up. Univariable and multivariable logistic regression models were developed to identify independent predictive risk factors for the aggravation of VS and SAS. Variables with *p* < 0.050 in univariable analysis were eligible for the inclusion as potential predictors into multivariable logistic regression analysis, although CRP level at > 10-year follow-up was excluded to establish predictive models. With regard to variables having two thresholds, e.g. age and CRP, to avoid overfitting, one with a lower odds ratio (OR) in univariable analysis was not included in multivariable analysis.

All statistical tests with statistical significance set at *p* values of < 0.050 and < 0.010 were performed using SPSS 28.0 (IBM Corp., Armonk, NY).

## Results

### Intraobserver and interobserver reliability

In quantitative measurements of cervical spine radiographs, the intraobserver reliability by ICC for parametric ADI, Ranawat value, subaxial vertebral translation, and C2–C7 angle was 0.91–0.95, 0.81–0.85, 0.80–0.84, 0.82–0.85 respectively. The interobserver reliability by ICC was 0.96, 0.91, 0.91, and 0.90, respectively. In qualitative assessments of hand radiographs, the intraobserver and interobserver reliability by κ for nonparametric Steinbrocker stages and mutilating changes was 0.75–0.81 and 0.87, respectively. All ICC and κ values indicated an acceptable reproducibility.

### Baseline demographic, clinical, and disease characteristics of dead or dropped-out patients at > 10-year follow-up

In this study cohort (*n* = 634), 86 (13.6%) were passed away while 315 (49.7%) were lost to follow-up during > 10 years. At baseline, the mean age of dead or dropped-out patients (*n* = 401) was 65.2 ± 11.3 years, which was older than 58.7 ± 9.7 years in the followed patients (*n* = 233) (*p* < 0.001). Then, 86 (21.4%) dead or dropped-out patients were male, whereas men in the followed patients were 34 (14.6%) (*p* = 0.034). While serum CRP level was 2.2 ± 2.8 mg/dL in the dead or dropped-out patients and 2.1 ± 2.6 mg/dL in the followed patients (*p* = 0.720), the distribution of Steinbrocker stages I–II, stages III–IV, and mutilating changes were 97 (24.2%), 246 (61.3%), and 58 (14.5%), respectively, in the dead or dropped-out patients versus 44 (18.9%), 167 (71.7%), and 22 (9.4%), respectively, in the followed patients (*p* = 0.027). Furthermore, the prevalence of pre-existing AAS, VS, and SAS in the dead or dropped-out patients was 138 (34.4%), 103 (25.7%), and 67 (16.7%), respectively, which were also higher than those of followed patients (AAS, 30.5%; VS, 19.3%; SAS, 7.7%) (*p* = 0.421). Factors associated with the study participation partially exists in demographic, clinical, and disease characteristics of patients.

### The prevalence of cervical spine instabilities during > 10 years

**Fig. 2:**
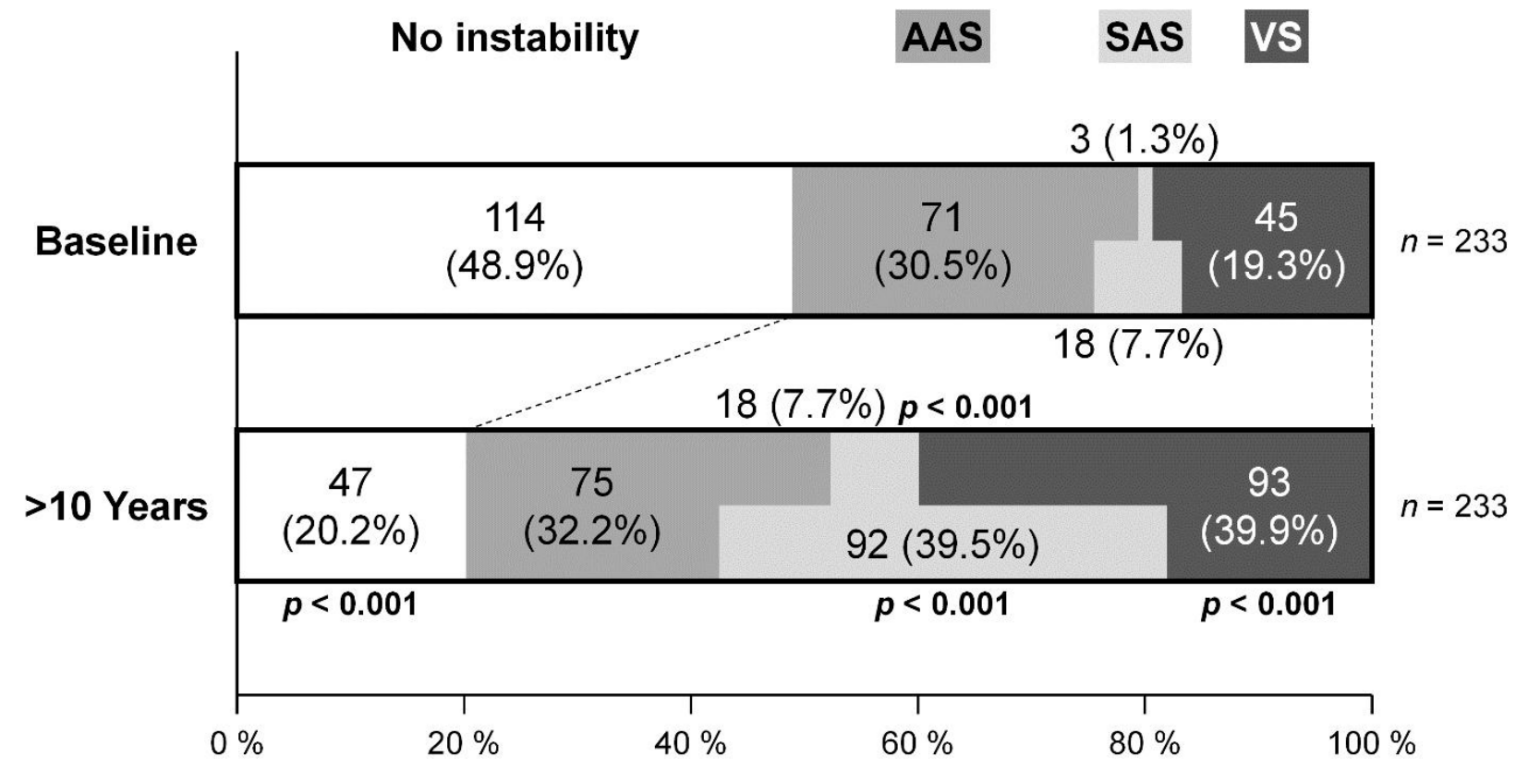
The prevalence of cervical spine instability comprising AAS, VS, and SAS at baseline and > 10-year follow-up in 233 patients. To simplify, VS combined with AAS was categorized as VS. The chi-squared test was used for the comparison.* AAS* atlantoaxial subluxation,* VS* vertical subluxation,* SAS* subaxial subluxation.

In 233 patients undergoing radiographic measurements at baseline and > 10-year follow-up, the number of those without any cervical spine instability decreased from 114 (48.9%) at baseline to 47 (20.2%) at endpoint (*p* < 0.001). The prevalence of AAS showed no statistically significant increase from 71 (30.5%) to 75 (32.2%) (*p* = 0.689). However, patients with VS significantly increased in number from 45 (19.3%) to 93 (39.9%) (*p* < 0.001). Patients with SAS alone also significantly increased from 3 (1.3%) to 18 (7.7%) (*p* < 0.001). Furthermore, SAS combined with or without other instabilities significantly increased from 18 (7.7%) to 92 (39.5%) (*p* < 0.001). Consequently, patients with at least one instability significantly increased from 119 (51.1%) to 186 (79.8%) (*p* < 0.001) (Fig. 2).

**Fig. 3:**
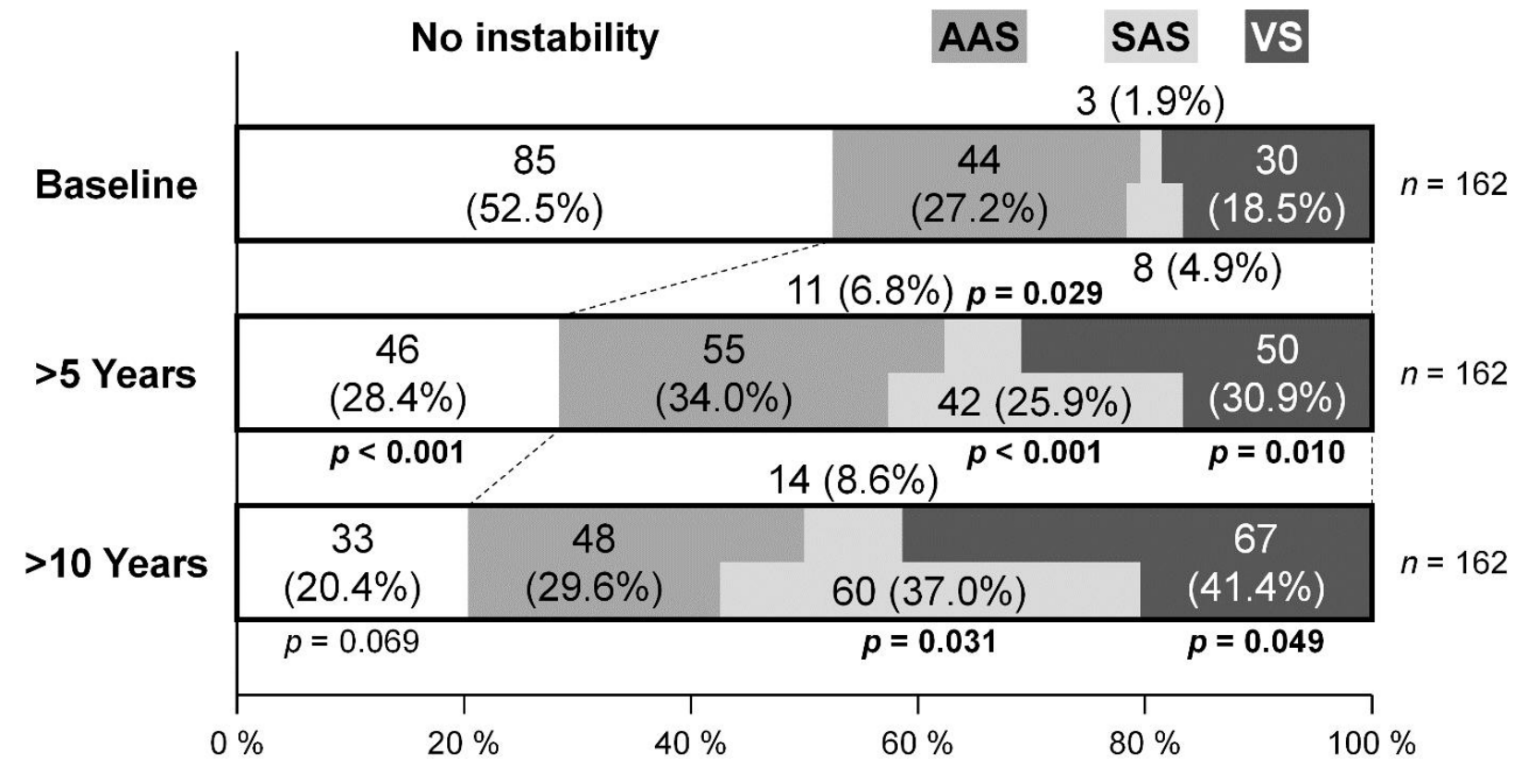
The prevalence of cervical spine instability comprising AAS, VS, and SAS at baseline, > 5-year follow-up, and > 10-year follow-up in 162 consecutively followed patients. To simplify, VS combined with AAS was categorized as VS. The chi-squared test was used for the comparison.* AAS* atlantoaxial subluxation,* VS* vertical subluxation,* SAS* subaxial subluxation.

In 162 patients undergoing consecutive radiographic measurements at baseline, > 5-year follow-up, and > 10-year follow-up, the number of those without any cervical spine instability significantly decreased from 85 (52.5%) to 46 (28.4%) during the initial > 5 years (*p* < 0.001), which further decreased to 32 (20.4%) during the last > 5 years but did not reach statistical significance (*p* = 0.069). The prevalence of AAS was 44 (27.2%) at baseline, 55 (34.0%) at > 5-year follow-up, and 48 (29.6%) at > 10-year follow-up, presenting no significant difference between both the initial and last > 5-year periods (*p* = 0.185 and *p* = 0.622, respectively). Meanwhile, VS significantly increased from 30 (18.5%) at baseline, 50 (30.9%) at > 5-year follow-up (*p* = 0.010), and consistently to 67 (41.4%) at > 10-year follow-up (*p* = 0.049). Also, SAS with or without other instabilities significantly increased from 8 (4.9%), 42 (25.9%) (*p* < 0.001), to 60 (37.0%) (*p* = 0.031) during > 10 years (Fig. 3).

### Predictive risk factors for the aggravation of VS

In 233 patients, the aggravation of VS was observed in 70 (30.0%) including 48 cases with newly developed VS and 22 cases with ≥ 2-mm decrease of the Ranawat value in pre-existing VS. When 45 patients without pre-existing VS were excluded (*n* = 188), 26 (36.6%) of 71 with pre-existing AAS experienced the aggravation of VS, while 22 (18.8%) of 117 without pre-existing AAS demonstrated the aggravation of VS (*p* = 0.007). Then, 22 (48.9%) of 45 with pre-existing VS further exhibited the aggravation of VS, whereas 48 (25.5%) of 188 without pre-existing VS displayed the aggravation of VS (*p* = 0.002). Similarly, 7 (38.9%) of 18 with pre-existing SAS had the trend toward the aggravation of VS compared to 63 (29.3%) of 215 without pre-existing SAS, although not reaching statistical significance (*p* = 0.394) (Table 1).

**Table 1:** The aggravation of VS and SAS based on pre-existing cervical spine instability in 233 patients at > 10-year follow-up.

Aggravated instability	Pre-existing instability			Aggravation		*p*
			*n*	*n*	%	
VS	AAS	(+)	71	26	36.6	0.007‡
		(−)	117	22	18.8	
	VS	(+)	45	22	48.9	0.002‡
		(−)	188	48	25.5	
	SAS	(+)	18	7	38.9	0.394
		(−)	215	63	29.3	
SAS	AAS	(+)	71	26	36.6	0.182
		(−)	117	32	27.4	
	VS	(+)	45	25	55.6	0.002‡
		(−)	188	58	30.9	
	SAS	(+)	18	9	50.0	0.184
		(−)	215	74	34.4	

Calculated by the chi-squared test.

*AAS* atlantoaxial subluxation,* SAS* subaxial subluxation,* VS* vertical subluxation.

‡*p* < 0.010.

In 162 consecutively followed patients, when 30 without pre-existing VS were excluded (*n* = 132), pre-existing AAS more frequently presented the aggravation of VS in 12 (27.3%) at > 5 years and 17 (38.6%) at > 10 years of 44 patients than 12 (12.8%) at > 5 years and 24 (25.5%) at > 10 years of 88 patients without pre-existing AAS (*p* = 0.030 and *p* = 0.097, respectively). Moreover, 14 (46.7%) at > 5 years and 16 (53.3%) at > 10 years of 30 patients with pre-existing VS showed the aggravation of VS, which were significantly higher than 20 (15.2%) at > 5 years and 37 (28.0%) at > 10 years of 132 patients without pre-existing VS (*p* < 0.001 and *p* = 0.008, respectively). However, pre-existing SAS was not significantly associated with the aggravation of VS both at > 5 and > 10 years (*p* = 0.674 and *p* > 0.999, respectively), possibly due to the small sample size (Supplementary Table S1).

In the comparison between patients with and without the aggravation of VS, those with VS aggravation tended to have a longer duration of RA (*p* = 0.010)—particularly ≥ 5 years (*p* < 0.001), higher level of CRP at baseline (*p* = 0.052)—especially ≥ 3.8 mg/dL (*p* = 0.009), higher frequency of previous peripheral joint surgery (*p* < 0.001), corticosteroid administration (*p* = 0.008), and mutilating changes in the hands (*p* < 0.001) at baseline. Male patients tended to be uncommon (*p* = 0.035). In addition, patients with CRP levels at > 10-year follow-up ≥ 3.8 mg/dL (*p* = 0.024) and > 1.0 mg/dL (*p* = 0.001) tended to develop the aggravation of VS (Table 2).

**Table 2:** Baseline and > 10-year demographic, clinical, and disease characteristics of 233 patients with and without the aggravation of VS at > 10-year follow-up.

	Patients with VS aggravation	Patients without VS aggravation	*p*
	(*n* = 70)	(*n* = 163)	
Age, mean ± SD years	58.6 ± 9.4	58.7 ± 9.8	0.918
<55 years, *n* (%)	21 (30.0)	56 (34.4)	0.806
55–64 years, *n* (%)	27 (38.6)	58 (35.6)	
≥65 years, *n* (%)	22 (31.4)	49 (30.1)	
Male sex, *n* (%)	5 (7.1)	29 (17.8)	0.035†
RA duration, mean ± SD years	16.3 ± 10.1	12.1 ± 10.8	0.010‡
≥5 years, *n* (%)	65 (92.9)	119 (73.0)	< 0.001‡
CRP, mean ± SD mg/dL at baseline	2.7 ± 2.9	2.0 ± 2.7	0.052
≥3.8 mg/dL, *n* (%)	20 (28.6)	23 (14.1)	0.009‡
>1.0 mg/dL, *n* (%)	43 (61.4)	93 (57.1)	0.535
CRP, mean ± SD mg/dL at > 10-year follow-up	2.0 ± 1.7	1.6 ± 3.2	0.314
≥3.8 mg/dL, n (%)	12 (17.1)	12 (7.4)	0.024†
>1.0 mg/dL, n (%)	47 (67.1)	72 (44.2)	0.001‡
RF positive, *n* (%)	59 (84.3)	132 (81.0)	0.548
Previous joint surgery, *n* (%)	47 (67.1)	66 (40.5)	< 0.001‡
Treatments			
Corticosteroids, *n* (%)	54 (77.1)	96 (58.9)	0.008‡
MTX, *n* (%)	35 (50.0)	63 (38.7)	0.108
Other DMARDs, *n* (%)	33 (47.1)	90 (55.2)	0.258
Biological agents, *n* (%)	8 (11.4)	11 (6.7)	0.231
Steinbrocker stages and mutilating changes			
Stages I–II, *n* (%)	6 (8.6)	38 (23.3)	< 0.001‡
Stages III–IV, *n* (%)	49 (70.0)	118 (72.4)	
Mutilating changes, *n* (%)	15 (21.4)	7 (4.3)	
C2–C7 angle	11.2 ± 14.9	13.4 ± 13.0	0.261

Calculated by the chi-squared test and student’s *t*-test.

*CRP* C-reactive protein,* DMARD* disease-modifying antirheumatic drug,* MTX* methotrexate,* RA* rheumatoid arthritis,* RF* rheumatoid factor,* SD* standard deviation.

†*p* < 0.050, ‡*p* < 0.010.

In an additional analysis of 45 patients with pre-existing VS, 5 (83.3%) of 6 patients with CRP level at >10-year follow-up ≥ 3.8 mg/dL developed the aggravation of VS, while 17 (43.6%) of 39 patients with CRP < 3.8 mg/dL developed the aggravation of VS (*p* = 0.070).

In logistic regression analysis, univariable models showed that the following variables had *p* < 0.050: mutilating changes (*p* < 0.001), previous joint surgery (*p* < 0.001), RA duration ≥ 5 years (*p* = 0.002), pre-existing VS (*p* = 0.003), corticosteroid administration (*p* = 0.009), and CRP level at baseline ≥ 3.8 mg/dL (*p* = 0.010). Male sex (*p* = 0.041) had a negative correlation with VS aggravation. To distinguish predictive risk factors for the aggravation of VS, CRP level at > 10-year follow-up was not included. Thereafter, we designed a multivariable logistic regression model including these variables, identifying mutilating changes (OR = 4.048, 95% confidence interval [CI] = 1.432–11.438, *p* = 0.008), RA duration ≥ 5 years (OR = 3.711, 95% CI = 1.279–10.769, *p* = 0.013), CRP level ≥ 3.8 mg/dL (OR = 2.187, 95% CI = 1.021–4.685, *p* = 0.044), and previous joint surgery (OR = 2.147, 95% CI = 1.122–4.108, *p* = 0.021) as significant independent predictive risk factors for the aggravation of VS (Table 3).

**Table 3:** Univariable and multivariable logistic regression models of 233 patients to identify predictive risk factors for the aggravation of VS at > 10-year follow-up.

	Univariable analysis			Multivariable analysis		
	OR	95% CI	*p*	OR	95% CI	*p*
< 55 years	0.886	0.490–1.603	0.689			
≥ 65 years	1.066	0.582–1.954	0.835			
Male sex	0.355	0.132–0.961	0.041†	0.477	0.166–1.373	0.170
RA duration ≥ 5 years	4.807	1.816–12.720	0.002‡	3.711	1.279–10.769	0.013†
CRP ≥ 3.8 mg/dL	2.435	1.233–4.809	0.010†	2.187	1.021–4.685	0.044†
CRP > 1.0 mg/dL	1.199	0.676–2.125	0.535			
RF positive	1.260	0.593–2.675	0.548			
Previous joint surgery	3.003	1.667–5.411	< 0.001‡	2.147	1.122–4.108	0.021†
Corticosteroids	2.355	1.243–4.464	0.009‡	1.679	0.832–3.391	0.148
MTX	1.587	0.902–2.792	0.109			
Other DMARDs	0.723	0.413–1.269	0.259			
Biological agents	1.783	0.684–4.645	0.236			
Steinbrocker stages III–IV	0.890	0.481–1.647	0.710			
Mutilating changes	6.078	2.354–15.690	< 0.001‡	4.048	1.432–11.438	0.008‡
Pre-existing AAS	1.549	0.855–2.807	0.149			
Pre-existing VS	2.790	1.427–5.453	0.003‡	1.449	0.675–3.109	0.341
Pre-existing SAS	1.535	0.569–4.141	0.397			
C2–C7 angle	0.988	0.967–1.009	0.262			

*AAS* atlantoaxial subluxation,* CI* confidence interval,* CRP* C-reactive protein,* DMARD* disease-modifying antirheumatic drug,* MTX* methotrexate,* OR* odds ratio,* RA* rheumatoid arthritis,* RF* rheumatoid factor,* SAS* subaxial subluxation,* VS* vertical subluxation.

†*p* < 0.050, ‡*p* < 0.010.

### Predictive risk factors for the aggravation of SAS

In 233 patients, the aggravation of SAS was shown in 83 (35.6%) including 74 without pre-existing SAS and 9 with pre-existing SAS, all of whom newly developed SAS at other segments and one additionally developed ≥ 2-mm increase of vertebral slippage in the segment of pre-existing SAS. Of 45 patients with pre-existing VS, 25 (55.6%) displayed the aggravation of SAS, while 58 (30.9%) of 188 without pre-existing VS demonstrated SAS aggravation (*p* = 0.002). Meanwhile, pre-existing AAS and SAS had the similar trend but did not reach any significant association with the aggravation of SAS (*p* = 0.182 and *p* = 0.184, respectively) (Table 1).

In 162 consecutively followed patients, 13 (43.3%) at > 5 years and 15 (50.0%) at > 10 years of 30 with pre-existing VS exhibited the aggravation of SAS, significantly higher than 23 (17.4%) at > 5 years and 41 (31.1%) at > 10 years of 132 patients without pre-existing VS (*p* = 0.002 and *p* = 0.049, respectively). On the other hand, pre-existing AAS and SAS was not significantly associated with the aggravation of SAS both at > 5 and > 10 years (*p* = 0.543–0.719 and *p* = 0.378–0.449, respectively) (Supplementary Table S1).

In the comparison between patients with and without the aggravation of SAS, those with SAS aggravation tended to have an older age (*p* = 0.073)—notably ≥ 65 years (*p* = 0.017), longer RA duration (*p* = 0.044), higher CRP level at baseline (*p* = 0.133)—especially ≥ 3.8 mg/dL (*p* = 0.045) and > 1.0 mg/dL (*p* = 0.003), higher frequency of previous joint surgery (*p* = 0.008), corticosteroid administration (*p* = 0.006), Steinbrocker stages III–IV or mutilating changes (*p* = 0.015), and larger C2–C7 angle (*p* = 0.073) at baseline. Similarly to VS aggravation, male patients tended to be uncommon (*p* = 0.048). Meanwhile, CRP level at > 10-year follow-up had no significant association with the aggravation of SAS (*p* = 0.509–>0.999) (Table 4).

**Table 4:** Baseline and > 10-year demographic, clinical, and disease characteristics of 233 patients with and without the aggravation of SAS at > 10-year follow-up.

	Patients with SAS aggravation	Patients without SAS aggravation	*p*
	(*n* = 83)	(*n* = 150)	
Age, mean ± SD years	60.2 ± 8.9	57.8 ± 10.0	0.073
<55 years, *n* (%)	18 (21.7)	59 (39.3)	0.017†
55–64 years, *n* (%)	33 (39.8)	52 (34.7)	
≥65 years, *n* (%)	32 (38.6)	39 (26.0)	
Male sex, *n* (%)	7 (8.4)	27 (18.0)	0.048†
RA duration, mean ± SD years	15.5 ± 11.1	12.3 ± 10.4	0.044†
≥5 years, *n* (%)	69 (83.1)	115 (76.7)	0.246
CRP, mean ± SD mg/dL at baseline	2.6 ± 2.5	2.0 ± 2.9	0.133
≥3.8 mg/dL, *n* (%)	21 (25.3)	22 (14.7)	0.045†
>1.0 mg/dL, *n* (%)	59 (71.1)	77 (51.3)	0.003‡
CRP, mean ± SD mg/dL at > 10-year follow-up	1.6 ± 1.6	1.8 ± 3.4	0.613
≥3.8 mg/dL, n (%)	10 (12.0)	14 (9.3)	0.509
>1.0 mg/dL, n (%)	42 (50.6)	77 (51.3)	> 0.999
RF positive, *n* (%)	69 (83.1)	122 (81.3)	0.732
Previous joint surgery, *n* (%)	50 (60.2)	63 (42.0)	0.008‡
Treatments			
Corticosteroids, *n* (%)	63 (75.9)	87 (58.0)	0.006‡
MTX, *n* (%)	33 (39.8)	65 (43.3)	0.597
Other DMARDs, *n* (%)	44 (53.0)	79 (52.7)	0.960
Biological agents, *n* (%)	9 (10.8)	10 (6.7)	0.265
Steinbrocker stages and mutilating changes			
Stages I–II, *n* (%)	8 (9.6)	36 (24.0)	0.015†
Stages III–IV, *n* (%)	64 (77.1)	103 (68.7)	
Mutilating changes, *n* (%)	11 (13.3)	11 (7.3)	
C2–C7 angle	14.9 ± 14.3	11.6 ± 13.1	0.073

Calculated by the chi-squared test and Student’s *t*-test.

*CRP* C-reactive protein,* DMARD* disease-modifying antirheumatic drug,* MTX* methotrexate,* RA* rheumatoid arthritis,* RF* rheumatoid factor,* SD* standard deviation.

†*p* < 0.050, ‡*p* < 0.010.

In logistic regression analysis, univariable models presented the following variables with *p* < 0.050: pre-existing VS (*p* = 0.002), CRP levels at baseline ≥ 3.8 mg/dL (*p* = 0.047) and > 1.0 mg/dL (*p* = 0.004), corticosteroid administration (*p* = 0.007), previous joint surgery (*p* = 0.008), and patients aged ≥ 65 years (*p* = 0.047). Patients aged < 55 years (*p* = 0.012) and male sex (*p* = 0.053) tended to have a negative correlation with SAS aggravation. To find predictors for the aggravation of SAS, CRP level at > 10-year follow-up was not analyzed. We therefore designed a multivariable logistic regression model including these variables, except for categorical variables with a lower OR in age (< 55 years) and CRP (≥ 3.8 mg/dL). This model identified pre-existing VS (OR = 2.252, 95% CI = 1.111–4.562, *p* = 0.024) and CRP > 1.0 mg/dL (OR = 2.139, 95% CI = 1.176–3.892, *p* = 0.013) as significant independent predictors for the aggravation of SAS (Table 5).

**Table 5:** Univariable and multivariable logistic regression models of 233 patients to identify predictive risk factors for the aggravation of SAS at > 10-year follow-up.

	Univariable analysis			Multivariable analysis		
	OR	95% CI	*p*	OR	95% CI	*p*
< 55 years	0.463	0.254–1.843	0.012†			
≥ 65 years	1.786	1.007–3.168	0.047†	1.786	0.971–3.287	0.062
Male sex	0.420	0.174–1.011	0.053			
RA duration ≥ 5 years	1.500	0.754–2.984	0.248			
CRP ≥ 3.8 mg/dL	1.971	1.008–3.853	0.047†			
CRP > 1.0 mg/dL	2.331	1.315–4.131	0.004‡	2.139	1.176–3.892	0.013†
RF positive	1.131	0.558–2.292	0.732			
Previous joint surgery	2.092	1.212–3.613	0.008‡	1.645	0.912–2.966	0.098
Corticosteroids	2.281	1.254–4.150	0.007‡	1.820	0.965–3.433	0.064
MTX	0.863	0.500–1.489	0.597			
Other DMARDs	1.014	0.593–1.735	0.960			
Biological agents	1.703	0.663–4.374	0.269			
Steinbrocker stages III–IV	1.537	0.829–2.850	0.172			
Mutilating changes	1.931	0.798–4.668	0.144			
Pre-existing AAS	1.558	0.908–2.672	0.107			
Pre-existing VS	2.802	1.442–5.445	0.002‡	2.252	1.111–4.562	0.024†
Pre-existing SAS	1.905	0.725–5.005	0.191			
C2–C7 angle	0.982	0.969–1.002	0.054			

*AAS* atlantoaxial subluxation,* CI* confidence interval,* CRP* C-reactive protein,* DMARD* disease-modifying antirheumatic drug,* MTX* methotrexate,* OR* odds ratio,* RA* rheumatoid arthritis,* RF* rheumatoid factor,* SAS* subaxial subluxation,* VS* vertical subluxation.

†*p* < 0.050, ‡*p* < 0.010.

## Discussion

This > 10-year prospective multicenter cohort study demonstrated time-course changes in the prevalence and aggravation of cervical spine instabilities in 2001–2013. Among the entire cohort followed at > 10 years (*n* = 233) and consecutive subcohort followed both at > 5 and > 10 years (*n* = 162), pre-existing AAS was associated with VS aggravation while pre-existing VS had a strong association with VS and SAS aggravation. Mutilating changes, RA duration ≥ 5 years, CRP level ≥ 3.8 mg/dL, and previous joint surgery were identified as independent predictive risk factors for the aggravation of VS. Pre-existing VS and CRP > 1.0 mg/dL were independent predictors for the aggravation of SAS.

The prevalence of cervical spine instabilities in RA varies, depending on multiple factors including disease duration, radiological criteria, follow-up period, and paradigm shifts in the treatment. Hence, many studies focused on changes in the prevalence and progression pattern of instabilities; however, prior investigations have been largely limited due to the cross-sectional or retrospective design with a shorter-period observation. Additionally, some studies concentrated only to AAS without evaluating VS and SAS^7,9^, which are more strongly associated with neurological dysfunction and even sudden death^13,34^. We studied longitudinal changes in the prevalence and progression pattern of cervical spine instabilities in patients with clinically relevant, symptomatic “classical” or “definite” RA during a relatively longer period of > 10 years. Among our cohort, the > 10-year prevalence of VS and SAS consistently increased, whereas AAS did not significantly increase. This trend could be explained by the two phenomena of AAS; the ADI increase as AAS aggravation and the ADI decrease as subclinical VS aggravation with the upward migration of the odontoid process by the destruction of the atlantoaxial facet joints^31,34,35^. We therefore focused on the aggravation of VS and SAS.

First, we clarified the association of VS and SAS aggravation with pre-existing cervical spine instability. Our prospective cohort studies at > 5-year follow-up (*n* = 267 and *n* = 228) clarified the association of VS and SAS aggravation with pre-existing VS and SAS^18,20^. A more recent 3.9-year retrospective follow-up study in the early biological agent era (*n* = 91) identified pre-existing cervical spine involvement as an independent predictive risk factor for the progression of instabilities^36^. Another 4.4-year retrospective follow-up study (*n* = 101) also demonstrated pre-existing AAS as a predictor for VS progression^23^. A 6.1-year different retrospective follow-up study in the era fully treated by biological agents (*n* = 87) found the association of pre-existing SAS with aggravated instabilities^37^. In the present study, pre-existing AAS without pre-existing VS was associated with the increased prevalence of VS. Notably, 48.9% in the entire cohort and 53.3% in the subcohort of patients with pre-existing VS (Ranawat value < 13 mm) developed ≥ 2-mm decrease of the Ranawat value during > 10 years. In VS, Ranawat value ≤ 10 mm is at risk of compression myelopathy^14,38,39^. Moreover, pre-existing VS was strongly associated with SAS aggravation. When surgically treating VS, spine surgeons should carefully consider the risk of SAS aggravation, which potentially leads to subaxial myelopathy.

Based on this study observation, larger C2–C7 angle, indicating the increase in cervical lordosis, could affect the aggravation of SAS relative to that of VS. Further studies are needed to clarify the involvement of the sagittal alignment in the pathomechanism of cervical spine instabilities.

Next, we examined the association of patients’ demographic, clinical, disease, and treatment characteristics with the aggravation of VS and SAS. Baseline longer RA duration, higher CRP level, previous joint surgery, corticosteroid administration, and mutilating changes were candidate predictive risk factors for the aggravation of VS. In addition to these variables, baseline age ≥ 65 years and Steinbrocker stages III–IV were also associated with the aggravation of SAS. Longer RA duration and higher CRP level at baseline imply the importance of low disease activity and clinical remission. This is in agreement with the current treat-to-target and early intervention strategy^40^. In fact, insufficient RA control with CRP level exceeding 1.0 mg/L at > 10-year follow-up was also associated with the aggravation of VS. Previous joint surgery and Steinblocker stages III–IV or mutilating changes reflect peripheral joint erosion and destruction, potentially collaborating with cervical spine involvement. Cervical spine instability was radiographically detected in 40.8–61.1% of patients with RA who underwent total hip or knee arthroplasty^30,41^. Advanced Steinblocker stages and mutilating changes are at higher risk of instability^19,21,26,28,31,32,35,42–44^. While concomitant corticosteroids are a known predictor of instability^6,7,13,45^, 10-mg/day prednisolone within 2 years increases the benefits of DMARDs and has joint-sparing properties in early RA^46^. A 4-year prospective study (*n* = 186) detected a lower dosage of oral corticosteroids as a risk factor of large joint destruction in the lower extremities^47^. Therefore, high disease activity requiring concomitant corticosteroids rather than corticosteroids themselves might impact on the development of cervical spine instabilities in RA.

Based on our multivariable logistic regression analysis, hand mutilating changes, RA duration ≥ 5 years, CRP level ≥ 3.8 mg/dL, and previous joint surgery were identified as independent predictive risk factors for the aggravation of VS. In addition, pre-existing VS and CRP > 1.0 mg/dL were also independent predictors for the aggravation of SAS. Interestingly, CRP is a common indicator of VS and SAS aggravation; however, the threshold was different. In a meta-analysis (*n* = 2750), CRP level was a predictive risk factor of cervical spine involvement^29^. Our findings of consistent association between no VS and SAS progression and CRP level < 1.0 mg/dL support CRP < 1.0 mg/dL as one of the reliable indicators for low disease activity and clinical remission^48^. Pre-existing VS was identified as a predictive risk factor for the aggravation of SAS but not VS. This might be due to the distribution of VS aggravation. Of 70 patients with the aggravation of VS during > 10-year follow-up, 22 (31.4%) with pre-existing VS developed ≥ 2-mm decrease of the Ranawat value and 48 (68.6%) without pre-existing VS developed the new onset of VS. The number of pre-existing VS might affect the statistical power in multivariable analysis.

Taken together, acquiring low disease activity before erosive joint destruction is essential to avert the > 10-year aggravation of cervical spine instabilities in RA, e.g. the introduction of biological agents. In this study, although the use of biological agents was not associated with no VS and SAS aggravation, it does not indicate no effectiveness of biological agents. As biological agents had been approved since 2003, 2 years after the 2001–2002 start of our surveillance in Japan, our results should be carefully interpreted. The incidence of cervical spine instabilities was still high at 31.8 to 41.8% in patients during the beginning era of biologic agents^42,43^. However, biological agents could prevent the new onset of cervical spine involvement in RA but fail to control the progression of pre-existing lesions^36,49^. In fact, a more recent cross-sectional study (*n* = 1333) reported the decreased prevalence of AAS by 50%, VS by 75%, and SAS by 5% in 2015, compared to in 1999^50^.

There are several limitations in this study. First, the follow-up rate was relatively low. In this study, 86 (13.6%) of 634 patients had died at 10-year follow-up. The comparison of baseline age, sex, RA severity, and cervical spine instability between followed patients versus dead or dropped-out patients indicate that many older, advanced RA outpatients could drop out due to polyarthralgia, worsening general condition, and/or cervical spine problems. Dropouts thus appear to be not randomly chosen in this study. Second, our data lacks clinical remission criteria such as the disease activity score-28 and magnetic resonance imaging findings, because of the study start in 2001–2002 and the last follow-up in 2012–2013. Moreover, our survey does not reflect the effect of more recent treatment options such as JAK inhibitors. Further investigation including these data would be desirable to validate the current study results.

In conclusion, this > 10-year prospective multicenter cohort study of 233 outpatients with RA elucidated the increased prevalence and aggravation of cervical spine instabilities, particularly VS and SAS, which was the most prominent in patients with pre-existing VS. Pre-existing VS itself, mutilating changes in the hands, RA duration ≥ 5 years, residual CRP level > 1.0 mg/dL—particularly ≥ 3.8 mg/dL, and previous peripheral joint surgery were predictive risk factors for further aggravation of instabilities. Low disease activity and clinical remission before the development of advanced peripheral joint erosion and destruction are essential to prevent progressive cervical spine instability in patients with RA.

## Electronic supplementary material

Below is the link to the electronic supplementary material.

Supplementary Material 1

## Data Availability

The datasets generated during and/or analyzed during the current study are not publicly available but are available from the corresponding author on reasonable request.
