# Supplementary material for: Prevalence and aggravation of cervical spine instabilities in rheumatoid arthritis during over 10 years: a prospective multicenter cohort study
Source: Sci Rep. 2024 Nov 5;14:26821. doi: 10.1038/s41598-024-78429-9 (PMC11538345; doi:10.1038/s41598-024-78429-9)
Supplement: Supplementary file 1 — Supplementary Material 1 [file 41598_2024_78429_MOESM1_ESM.pdf]

SUPPLEMENTARY INFORMATION

Supplementary Table S1. The aggravation of VS and SAS based on pre-exiting cervical spine instability in 162 consecutively followed patients both at >5-year and >10-year follow-ups.

Aggravated instability	Pre-exiting instability			Aggravation at >5 years			Aggravation at >10 years		
			n	*n*	%	*p*	*n*	%	*p*
VS	AAS	(+)	44	12	27.3	0.030†	17	38.6	0.097
		(−)	88	12	12.8		24	25.5	
	VS	(+)	30	14	46.7	<0.001‡	16	53.3	0.008‡
		(−)	132	20	15.2		37	28.0	
	SAS	(+)	8	2	25.0	0.674*	2	25.0	>0.999*
		(−)	154	32	20.8		51	33.1	
SAS	AAS	(+)	44	8	18.2	0.719	16	36.4	0.543
		(−)	88	20	21.3		30	31.9	
	VS	(+)	30	13	43.3	0.002‡	15	50.0	0.049†
		(−)	132	23	17.4		41	31.1	
	SAS	(+)	8	3	37.5	0.378*	4	50.0	0.449*
		(−)	154	33	21.4		52	33.8	

Calculated by the chi-squared test (Fisher's exact test* when $n \leq 5$ in a cell).

†$p < 0.050$, ‡$p < 0.010$.

AAS, atlantoaxial subluxation; SAS, subaxial subluxation; VS, vertical subluxation.
